# Quality of dietary macronutrients is associated with glycemic outcomes in adults with cystic fibrosis

**DOI:** 10.3389/fnut.2023.1158452

**Published:** 2023-09-20

**Authors:** Tanicia C. Daley, Benjamin A. Cousineau, Paula-Dene C. Nesbeth, Elizabeth A. Ivie, Moriah P. Bellissimo, Kirk A. Easley, Priyathama Vellanki, Miriam B. Vos, William R. Hunt, Arlene A. Stecenko, Thomas R. Ziegler, Jessica A. Alvarez

**Affiliations:** ^1^Department of Pediatrics, Emory University School of Medicine, Atlanta, GA, United States; ^2^Department of Medicine, Emory University School of Medicine, Atlanta, GA, United States; ^3^Nutrition and Health Sciences Doctoral Program, Laney Graduate School, Emory University, Atlanta, GA, United States; ^4^Department of Internal Medicine, Division of Cardiology, Virginia Commonwealth University School of Medicine, Richmond, VA, United States; ^5^Department of Biostatistics and Bioinformatics, Rollins School of Public Health, Emory University School of Medicine, Atlanta, GA, United States

**Keywords:** cystic fibrosis, diabetes, diet quality, insulin secretion, insulin resistance, glucose tolerance, nutrition

## Abstract

**Objective:**

Poor diet quality contributes to metabolic dysfunction. This study aimed to gain a greater understanding of the relationship between dietary macronutrient quality and glucose homeostasis in adults with cystic fibrosis (CF).

**Design:**

This was a cross-sectional study of *N* = 27 adults with CF with glucose tolerance ranging from normal (*n* = 9) to prediabetes (*n* = 6) to being classified as having cystic fibrosis-related diabetes (CFRD, *n* = 12). Fasted blood was collected for analysis of glucose, insulin, and C-peptide. Insulin resistance was assessed by Homeostatic Model Assessment for Insulin Resistance (HOMA2-IR). Subjects without known CFRD also underwent a 2-h oral glucose tolerance test. Three-day food records were used to assess macronutrient sources. Dietary variables were adjusted for energy intake. Statistical analyses included ANOVA, Spearman correlations, and multiple linear regression.

**Results:**

Individuals with CFRD consumed less total fat and monounsaturated fatty acids (MUFA) compared to those with normal glucose tolerance (*p* < 0.05). In Spearman correlation analyses, dietary glycemic load was inversely associated with C-peptide (rho = −0.28, *p* = 0.05). Total dietary fat, MUFA, and polyunsaturated fatty acids (PUFA) were positively associated with C-peptide (rho = 0.39–0.41, all *p* < 0.05). Plant protein intake was inversely related to HOMA2-IR (rho = −0.28, *p* = 0.048). Associations remained significant after adjustment for age and sex.

**Discussion:**

Improvements in diet quality are needed in people with CF. This study suggests that higher unsaturated dietary fat, higher plant protein, and higher carbohydrate quality were associated with better glucose tolerance indicators in adults with CF. Larger, prospective studies in individuals with CF are needed to determine the impact of diet quality on the development of CFRD.

## Introduction

Cystic fibrosis (CF) is an autosomal recessive genetic disease that leads to multi-organ impairment, including the lungs, pancreas, and gastrointestinal system (1). The disease results from cystic fibrosis transmembrane conductance regulator (CFTR) protein defects resulting in the inability to transport chloride and bicarbonate across epithelial cell membranes, subsequent mucosal abnormalities, and downstream chronic inflammation and tissue damage. Cystic fibrosis-related diabetes (CFRD) is one of the most common co-morbidities of CF and is associated with significantly increased morbidity and mortality (2). It is currently proposed that a decline in β-cell function begins in early childhood and results in diminished insulin secretion and delayed first phase insulin response (2, 3). In addition, individuals with CF have a diminished incretin response that contributes to impaired insulin secretion (4). Secondary factors (either intrinsic or extrinsic) may hasten the development of CFRD. Some of these factors are known, such as having first degree relatives with type 2 diabetes and having pancreatic exocrine insufficiency (5). Whether there are modifiable secondary factors contributing to CFRD development, such as dietary intake, is unknown.

The CF diet is typically high in energy-dense, nutrient-poor foods. Historically, individuals with CF were prescribed high-energy, high-fat diets to maintain their hypermetabolic state and offset malabsorption. Current CF dietary guidelines recommend an energy intake of 1.2 to 1.5 times that of the general population (6), but many in the CF community are revisiting the validity of this recommendation and there are growing calls to formulate evidence based dietary guidelines. Many people living with CF consume a diet abundant in high glycemic index (GI) foods, sugar sweetened beverages (SSBs), and refined sugars (6, 7). Further, the clinical recommendation for a high-fat diet in individuals with CF has historically resulted in an over-reliance on dietary saturated fats (7–9). Dietary intake analyses of individuals with CF reflect diets that are generally low in quality, as indicated by a disproportionately high intake of energy-dense, yet nutrient-poor, foods in children (7) and high added sugar and trans-fatty acid intake, as well as low Healthy Eating Index scores, in adults (10).

In non-CF populations, prolonged consumption of excess added sugars and saturated fat appear to promote a decline in β-cell function and increase insulin resistance (11–14). Although data are mixed, many studies suggest that fat quality is important in preventing the onset of type 2 diabetes (15). More specifically, diets high in saturated fat are associated with reduced insulin secretion, whereas diets high in monounsaturated fatty acids (MUFA) are associated with enhanced insulin secretion (15). Increased consumption of MUFA and polyunsaturated fatty acids (PUFA) can also lead to a reduction in HbA1C and insulin resistance (15–17). While dietary protein has known insulinotropic effects (18), observational studies in non-CF populations have indicated increased risk for diabetes with higher consumption of animal proteins and lower or no diabetes risk with higher plant protein intake (19, 20). The impact of dietary sources of macronutrients on glucose homeostasis for individuals with CF is unknown.

To date, there is a paucity of studies that investigate the metabolic sequelae of poor diet quality for individuals with CF. Thus, the purpose of this study was to quantify the relationships of diet quality indicators at the macronutrient level (e.g., carbohydrate, fat, protein) with measures of glucose homeostasis in adults with CF across the glucose tolerance spectrum. Such data may guide evidence-based updates to dietary recommendations for people living with CF.

## Methods

### Materials and methods

#### Subjects and study design

This was a prospective, cross-sectional study, which included 27 adults with clinically stable CF enrolled between 2014 and 2018. Details about the study were previously reported for a sub-set of participants and, in comparison, healthy controls (10). Briefly, inclusion criteria were, for those with CF, a confirmed CF diagnosis via chloride sweat test and/or CFTR genetic test with at least one Class I, II, or III CFTR mutation. All individuals with CF had pancreatic exocrine insufficiency and received pancreatic enzyme replacement therapy. For testing, participants were required to be on a stable medical regimen, including no oral or intravenous antibiotics or glucocorticoids, for at least 3 weeks. Exclusion criteria were current pregnancy, inability to fast overnight (including enteral tube feeds), and the most recent forced expiratory volume in 1 s (FEV1) expressed as a percentage of the predicted value (FEV1%) of <40%. All testing was performed following an overnight fast within the Georgia Clinical and Translational Science Institute Emory University Hospital Clinical Research Center (GCRC). Height and weight were assessed within the GCRC for determination of body mass index (BMI). Most recent lung function reported as percent predicted forced expiratory volume in one second (FEV1% predicted), was extracted from the electronic medical record based on spirometry performed at the Emory University Hospital Adult CF Clinic. The study was approved by the Emory Institutional Review Board, and written informed consent was conducted before any testing.

#### Assessment of glucose metabolism

For participants without previously diagnosed CFRD (*n* = 18), a standard 2-h oral glucose (75 g) tolerance test (OGTT) was performed and glucose tolerance status (normal glucose tolerance, pre-diabetes, or CFRD) was determined using cut-off glucose values recommended by the American Diabetes Association (21). For participants who already had a clinical diagnosis of CFRD within the medical chart (*n* = 9), short-acting insulin was not taken in the morning, as applicable, and only fasted blood was drawn. Blood was collected and processed on the day of the study. Serum was stored at −80°C until ready for analysis. Serum glucose concentrations (fasted and 2-h) were determined in real time in the Emory University Hospital Clinical Laboratory using a standard enzymatic assay for clinical care. Fasted serum insulin and C-peptide were assayed at the University of Alabama-Birmingham Metabolism Core in paired replicates using an immunofluorescence assay (TOSOH AIA 900, TOSOH Bioscience, South San Francisco, CA). The assay does not distinguish between types of insulin. The inter-assay (variation of controls between assays) and intra-assay (variation of replicates within the same assay) coefficients of variation within the Core are 3.95 and 1.49% for insulin and 6.81 and 1.67% for C-peptide, respectively. Insulin resistance was calculated with fasted glucose and insulin levels using the updated computer Homeostatic Model Assessment of Insulin Resistance (HOMA2-IR) (22).

#### Dietary intake

Participants were provided detailed instructions for completion of a three-day food record to include two consecutive weekdays and one weekend day. On receipt of the food record, a registered dietitian reviewed the record with the participant and asked probing questions for missed details. Records were analyzed for total kilocalories (kcal) and macronutrient composition using the Nutrition Data System for Research software (NDSR, Nutrition Coordinating Center, University of Minnesota, MN, USA; database version 2018). Dietary intake information was previously reported for a sub-set of participants (10). The current analysis focused on assessment of macronutrients from varying sources (e.g., saturated fats vs. monounsaturated fats, plant vs. animal protein intake). Dietary information was not available from one participant.

#### Statistical analyses

Descriptive statistics were performed on all variables. To account for differences of total caloric intake, dietary variables (except total kcal and glycemic index) were adjusted per 1,000 kcal for statistical analyses. Kruskal-Wallis tests (for continuous variables) or Fischer’s exact test (for categorical variables) were used to compare variables between CF participants across the glucose tolerance groups. Post-hoc Steel-Dwass nonparametric multiple comparison tests were performed for variables as indicated (23). Associations between dietary intake variables and glucose tolerance outcomes were analyzed using Spearman rank correlations. Relationships were subsequently assessed using multiple linear regression, with adjustment for age and sex, and with log-transformed outcomes variables as needed based on visual inspection of a normal distribution. All analyses were conducted in JMP® Pro software version 15.0.0 (SAS Institute, Cary, NC), using two-sided tests with an alpha significance value of 0.05.

## Results

Demographic information of the *N* = 27 study participants with CF is provided in Table 1. Briefly, the mean age was 26 years. Approximately half the cohort was female (51%), and approximately half (52%) were homozygous for the delF508 mutation. The mean BMI (21.4 kg/m^2^) was below target recommendations (10, 24). Participants had moderate lung disease based on mean FEV1 (75% predicted). A total of 44% of participants had CFRD, 22% had pre-diabetes, and 33% had normal glucose tolerance. Table 2 provides demographic information based on glucose tolerance status. CFRD was more prevalent in females compared to males (75% vs. 25%, *p* = 0.05). Fasting glucose, insulin, or HOMA2-IR did not significantly differ between glucose tolerance groups (all *p* > 0.05, Table 3). Nine participants with CFRD were being treated with exogenous insulin for glucose control, three of whom were on a basal insulin regimen. Post-hoc exclusion of three participants on a basal insulin regimen did not alter the results. As expected, individuals with CFRD had significantly lower fasting C-peptide compared to those with normal glucose tolerance (*p* < 0.05), and those with pre-diabetes and CFRD had significantly higher 2-h glucose values following the OGTT compared to those with normal glucose tolerance (*p* < 0.05).

**Table 1:** Demographics of adults with cystic fibrosis (*n* = 27).

	Median (IQR) or *n* (%)
Age	26.3 (21.5, 34.6)
Sex	
Female	14 (51%)
Male	13 (48%)
Race^1^	
White/Caucasian	23 (85%)
Black/African American	4 (15%)
Genotype	
delF508 homozygous	14 (52%)
delF508 heterozygous	12 (44%)
Other	1 (4%)
BMI (kg/m^2^)	20.9 (19.7, 23.9)
FEV1(% predicted)	75 (61, 85)
Glucose tolerance	
Normal glucose tolerance	9 (33%)
Pre-diabetes	6 (22%)
CF-related diabetes	12 (44%)

BMIbody mass index; FEV1forced expiratory volume in 1 s. ^1^*n* = 1 Hispanic Caucasian.

**Table 2:** Demographics by glucose tolerance status.

	Normal glucose tolerance (*n* = 9)	Pre-diabetes (*n* = 6)	CFRD (*n* = 12)	*p*-value^a^
Age (years)	22.5 (20.3, 31.1)	26.4 (21.6, 37.7)	27.7 (21.2, 38.5)	0.31
Sex [*n* (%)]				0.05
Female	2 (22%)	3 (50%)	9 (75%)	
Male	7 (78%)	3 (50%)	3 (25%)	
Race [*n* (%)]				0.80
White	7 (78)	5 (83)	11 (92)	
Black	2 (22)	1 (17)	1 (8)	
delF508 homozygous [*n* (%)]	6 (67%)	2 (50%)	5 (42%)	0.84
BMI (kg/m^2^)	20.7 (17.9, 24.5)	22.1 (20.3, 23.5)	21.3 (19.2, 24.3)	0.81
FEV1 (%predicted)	75.0 (57.5, 102.0)	78.5 (71.3, 82.0)	68.5 (53.5, 84)	0.58

Reported as median (IQR) or *n* (%). ^a^Determined by Kruskal-Wallis test for continuous variables or Fischer’s exact test for categorical variables. BMI, body mass index; FEV1, forced expiratory volume in 1 s.

**Table 3:** Biochemical variables by glucose tolerance status^1^.

	Normal Glucose Tolerance (*n* = 9)	Pre-Diabetes (*n* = 6)	CFRD (*n* = 12)	*p*-value
Fasting glucose (mg/dL)	88 (76, 95)	93 (80, 107)	98 (77, 107)	0.44
Fasting insulin (uIU/mL)	5.6 (3.7, 6.2)	4.7 (2.0, 6.3)	3.6 (2.2, 6.7)	0.60
Fasting C-peptide (ng/mL)	1.09 (0.92, 1.41)	1.0 (0.6, 1.4)	0.50 (0.18, 0.81)^3^	**0.02**
2-h glucose^2^	100 (62, 124)	148 (136, 171)^3^	214 (202, 222)^3^	**0.002**
HOMA2-IR	0.6 (0.4, 0.7)	0.5 (0.3, 0.7)	0.4 (0.2, 0.7)	0.74

^1^Reported as median (IQR). For each variable with value of *p* <0.05, groups not connected by the same letter significantly differ via Steel-Dwass nonparametric comparison test. CFRD, CF-related diabetes; HOMA2-IR, homeostatic assessment of insulin resistance-2. Bold values indicate statistical significance (*p* < 0.05). ^2^*n* = 9 NGT, 6 pre-diabetes, and 3 CFRD. ^3^Statistically significantly different compared to the normal glucose tolerance group, as determined by Steel-Dwass nonparametric comparison tests.

### Dietary intake by glucose tolerance status

Dietary intake information by glucose tolerance status is provided in Table 4. Both total fat intake and MUFA intake were significantly lower in those with CFRD compared to those with normal glucose tolerance (*p* = 0.005 and 0.02, respectively). Total kcal, total dietary carbohydrates, added sugars, or dietary protein intake did not differ significantly by glucose tolerance group (all *p* > 0.05).

**Table 4:** Dietary variables by glucose tolerance status^1^.

	Normal glucose tolerance (*n* = 8)	Pre-diabetes (*n* = 6)	CFRD (*n* = 12)	*p*-value
Total calories (kcal)	3,188 (2,596, 4,020)	2,663 (2,586, 4,094)	2,279 (1992, 2,941)	0.08
Total carbohydrates (g)	343.8 (285.6, 436.1)	383.9 (253.9, 432.1)	276.0 (258.4, 354.9)	0.10
Added sugars (g)	79.8 (40.0, 89.6)	84.9 (71.4, 144.5)	102.7 (44.5, 140.3)	0.06
Glycemic index	62.3 (60.8, 66.6)	64.1 (57.7, 65.8)	62.4 (56.4, 65.3)	0.79
Glycemic load	206.5 (152.6, 269.0)	226.3 (136.3, 263.0)	171.6 (130.4, 215.4)	0.24
Total fat (g)	138.1 (110.8, 173.4)	122.1 (98.3, 163.2)	86.1 (63.5, 102.3)^2^	**0.005**
Saturated fat (g)	46.6 (30.2, 63.3)	39.8 (32.5, 59.7)	29.7 (19.8, 38.8)	0.14
Trans-fat (g)	3.1 (2.0, 4.9)	3.0 (1.6, 6.1)	2.0 (1.6, 4.5)	0.91
MUFA (g)	55.3 (37.9, 56.8)	44.8 (29.8, 55.2)	25.2 (21.7, 35.9)^2^	**0.02**
PUFA (g)	27.4 (21.8, 35.7)	33.0 (21.4, 39.7)	19.5 (15.5, 23.0)	0.07
Total protein (g)	145.8 (103.8, 170.8)	119.7 (85.3, 168.8)	97.7 (90.5, 125.3)	0.31
Animal protein (g)	107.4 (51.6, 131.4)	88.5 (48.6, 134.1)	76.3 (47.5, 96.5)	0.71
Plant protein (g)	44.9 (31.1, 58.9)	35.3 (24.9, 43.9)	22.5 (19.8, 31.0)	0.38

^1^Reported as median (IQR). Dietary variables (except total kcal and glycemic index) are adjusted per 1,000 kcal for statistical analyses. For each dietary variable, groups not connected by the same letter significantly differ via Steel-Dwass nonparametric comparison test. *N* = 26 as dietary data was missing for one subject. Bold values indicate statistical significance (*p* < 0.05). ^2^Statistically significantly different compared to the normal glucose tolerance group, as determined by Steel-Dwass nonparametric comparison tests.

### Association between dietary intake and glucose homoeostasis measures

**Figure 1:**
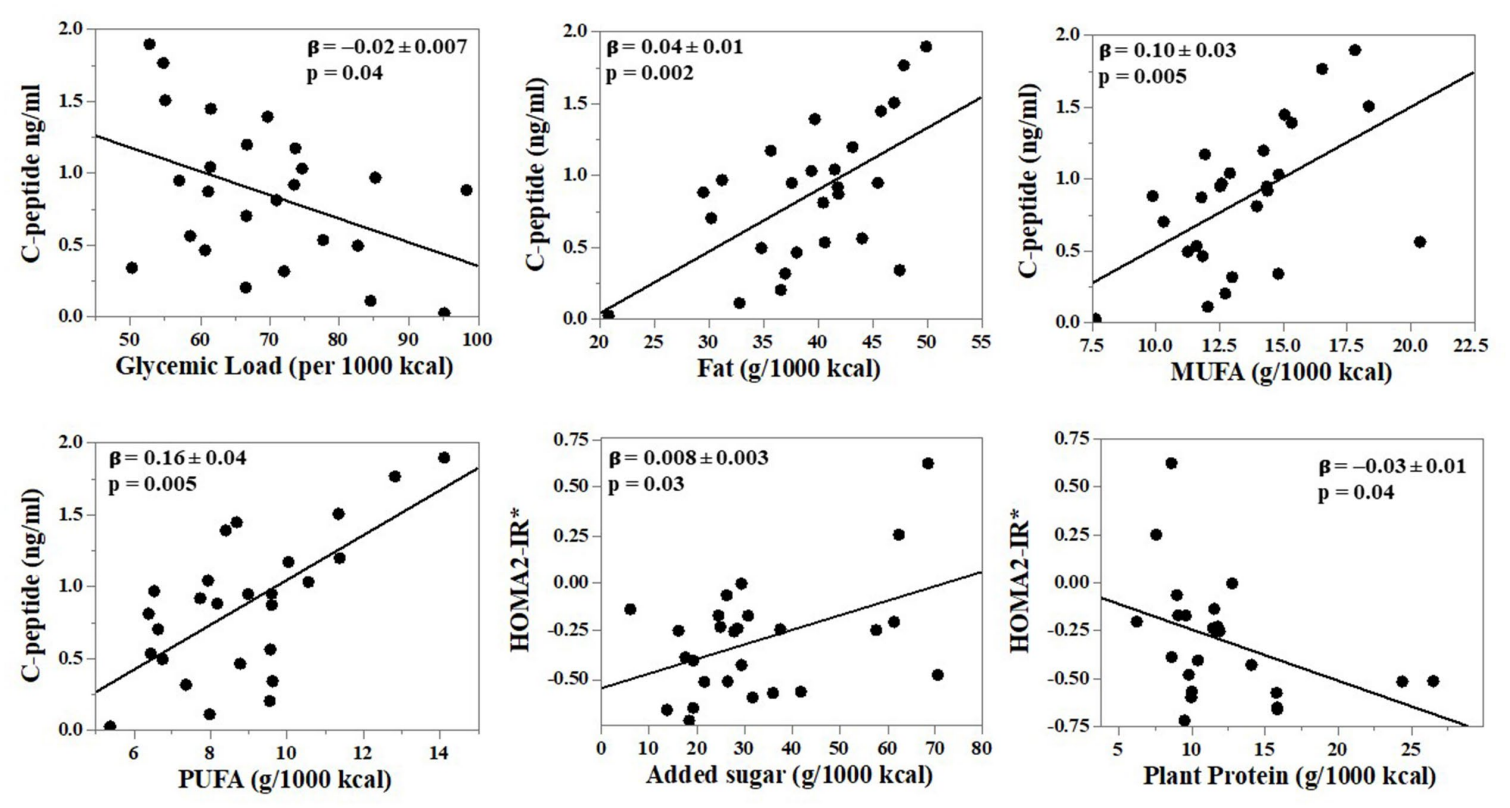
Independent relationships between nutrients reflecting diet quality and glucose tolerance outcomes in adults with cystic fibrosis (CF). *N* = 26 adults with CF. Dietary intake was collected via 3-day food records and analyzed using Nutrition Data Systems for Research. Multiple linear regression (MLR) analyses were performed with adjustment for age and sex. Shown are relationships that were statistically significant in both Spearman correlation and MLR analyses. Results of additional MLR analysis are provided in Supplementary Table S1. The asterisk in figure with HOMA2-IR =*Log10-transformed for analyses.

Fasting glucose or fasting insulin did not significantly correlate with the dietary variables (Table 5). Fasting C-peptide concentrations were inversely associated with total carbohydrate intake and dietary glycemic load (rho = −0.28, *p* = 0.05 for both). Fasting C-peptide was significantly, positively associated with total dietary fat (rho = 0.41, *p* = 0.003), MUFA (rho = 0.40, *p* = 0.004) and PUFA (rho = 0.39, *p* = 0.006). There was a possible association between HOMA2-IR and added sugar intake (rho = 0.25, *p* = 0.08) although it did not reach statistical significance, and it was inversely associated with plant protein intake (rho = −0.28, *p* = 0.048). Relationships were similar after adjusting for age and sex using multiple linear regression (MLR) modeling (Supplementary Table S1). Of note, the inverse relationship between fasting C-peptide concentrations and total carbohydrate intake and dietary glycemic load became statistically significant after adjusting for age and sex (*β* = −0.01 ± 0.005, *p* = 0.01 and *β* = −0.02 ± 0.007, *p* = 0.04, respectively). The relationship between HOMA2-IR and added sugar intake likewise became statistically significant (*β* = 0.008 ± 0.003, *p* = 0.03). After adjustment for age and sex, statistically significant inverse relationships of 2-h glucose with total fat and MUFA emerged (*β* = −5.6 ± 1.86, *p* = 0.01 and *β* = −9.51 ± 4.32, *p* = 0.046, respectively). Regression plots for relationships that were similar in both Spearman and MLR analyses are shown in Figure 1. Results were similar after post-hoc exclusion of three participants on a basal insulin regimen.

**Table 5:** Spearman correlations between dietary variables and glucose tolerance outcomes^1^.

Dietary variable^2^	Fasting glucose (mg/dL)	Fasting insulin (uIU/mL)	Fasting C-peptide (ng/mL)	2-h glucose^3^	HOMA2-IR
Total CHO (g)	0.13 (0.35)	0.08 (0.58)	−0.28 (0.05)	0.04 (0.80)	0.06 (0.67)
Added sugars (g)	0.23 (0.11)	0.23 (0.10)	**−**0.19 (0.19)	0.28 (0.08)	0.25 (0.08)
Glycemic index	0.05 (0.74)	0.09 (0.54)	**−**0.15 (0.30)	**0.33 (0.04)**	0.10 (0.49)
Glycemic load	0.13 (0.37)	0.13 (0.35)	−0.28 (0.05)	0.22 (0.18)	0.13 (0.37)
Total fat (g)	0.05 (0.71)	0.22 (0.12)	**0.41 (0.003)**	−0.006 (0.99)	0.20 (0.15)
Saturated fat (g)	0.07 (0.65)	0.18 (0.19)	0.22 (0.13)	0.02 (0.91)	0.15 (0.30)
Trans-fat (g)	0.09 (0.54)	**−**0.06 (0.69)	0.05 (0.72)	0.29 (0.07)	−0.05 (0.72)
MUFA (g)	**−**0.03 (0.82)	0.03 (0.86)	**0.40 (0.004)**	0.03 (0.88)	0.04 (0.79)
PUFA (g)	0.02 (0.91)	0.21 (0.13)	**0.39 (0.006)**	0.02 (0.90)	0.21 (0.14)
Total protein (g)	**−**0.20 (0.15)	**−**0.13 (0.37)	**−**0.03 (0.84)	**−**0.07 (0.67)	**−**0.12 (0.42)
Animal protein (g)	**−**0.03 (0.82)	0.007 (0.96)	0.007 (0.96)	**−**0.02 (0.90)	0.02 (0.89)
Plant protein (g)	**−**0.19 (0.18)	**−**0.28 (0.05)	0.04 (0.79)	−0.25 (0.12)	**−0.28 (0.048)**

^1^Reported as Spearman’s rho (*p*-value). Bold values indicate statistical significance (*p* < 0.05). ^2^Dietary variables are adjusted per 1,000 kcal. ^3^*N* = 17.

## Discussion

The present study provides an analysis of the typical diet of adults with CF across glucose spectrum to determine whether dietary intake, with special attention paid to the nutrient source and diet quality of macronutrients, is associated with unfavorable outcomes for glucose homeostasis. We found that the source of macronutrients, including added sugars and glycemic load, MUFA, and plant protein intake, were significant correlates of glucose tolerance indicators, drawing attention to the importance of diet quality.

Studies investigating diet quality in CF, have focused on carbohydrate quality in the form of sugar sweetened beverages, glycemic index, or glycemic load (7, 10). Sutherland et al. found that children with CF consumed twice as many calories from sweetened drinks, confectionary sugars and packaged snacks when compared to control children (7), resulting in a diet high in added sugars and saturated fats with very sub-optimal micronutrients in the diet (7). We previously reported that adults with CF consumed large amounts of added sugar compared to controls, and this was associated with increased visceral adipose tissue (10), a risk factor for insulin resistance. In this updated analysis, the median total added sugar intake (83.2 g, 12.6% of total kcal daily) was above the recommended limits suggested by the American Heart Association and the 2020 Dietary Guidelines for Americans (<6 and < 10% of total kcal intake, respectively) (10, 25).

Further, we found a significant positive relationship between added sugar intake and insulin resistance and a significant inverse relationship between dietary glycemic load and fasting C-peptide, independent of age and sex. The role of chronic high glycemic load intake on insulin secretion in people with CF is unknown, but it is possible that chronic glucose stimulation by high carbohydrate, high glycemic load diets induces glucose toxicity to pancreatic β-cells (26). In another adult CF study, added sugar intake and glycemic load significantly correlated with higher glucose variability and less time in the euglycemic range during continuous glucose monitoring (27). Whether interventions to modify carbohydrate quality influence glucose tolerance in adults with CF is unknown, although a low glycemic index behavioral intervention in a pediatric CF cohort decreased, albeit not statistically significantly, fasting glucose levels (28). We did not find significant relationships between dietary variables and fasting glucose; however, fasting glucose is not a hallmark feature of CFRD (29), and it has been hypothesized that people with CF have enhanced glucose utilization (30).

Aside from achieving a goal of 35–40% of calories consumed from fats, there have historically been no recommended restrictions on the type of fats to consume in the CF diet. Like added sugars, the clinical recommendation for a high-fat diet in individuals with CF has resulted in an over-reliance on dietary saturated fats (7–9), in agreement with results of this study showing a median saturated fat intake of 12% of total kcal. Less emphasis in adults with CF has been placed on intake of unsaturated fats (MUFA and PUFA). Current study findings revealed positive correlations between fasted C-peptide concentrations and MUFA and PUFA. In non-CF populations, increased consumption of unsaturated fatty acids may improve glucose homeostasis (17, 31), with human acute meal challenges showing insulin secretory effects of MUFA through the action of the incretin glucagon-like peptide-1 (GLP-1) (32–34). The links between unsaturated fat consumption and glucose homeostasis generated evidence-based dietary guidelines for the general population that suggest diets high in vegetables, vegetable oils, nuts, and fish can decrease the risk of developing type 2 diabetes (17). In models adjusted for age and sex, the two-hour serum glucose level was also inversely correlated with MUFA intake. While further dietary interventions and meal challenge testing is required in adults with CF, these data suggest general population recommendations for increased intake of unsaturated fatty acids with concomitant decrease in saturated fatty acids (35), should apply to adults with CF.

We found a novel inverse relationship between HOMA-IR and plant protein intake among participants with CF. Plant proteins, compared to animal proteins, have previously been shown to correlate with a reduced prevalence or risk of developing type 2 diabetes (36, 37) and lower fasting insulin and glucose in populations with type 2 diabetes (38). Likewise, some, but not all (39), plant-based intervention studies in overweight adults have shown improvements in β-cell function and insulin sensitivity (40). Whether plant protein has a direct effect on insulin action, is not clear, as several nutrients commonly found in plant foods may play a role in mitigating the effects of insulin resistance, including polyphenols, such as genistein (41). Plant-based dietary interventions for glycemic control have not been studied in CF populations, likely owing to the historical emphasis on consumption of energy-dense foods.

Our study suggests that modifications to the typical CF diet, with decreased consumption of added sugars and increased consumption of MUFA, PUFA, and plant proteins, may improve glucose tolerance in adults with CF. As the lifespan of individuals with CF continues to increase, understanding the long-term sequelae of an unrestricted diet is of upmost importance for individuals living with CF. Evidence-based research in diet quality is gaining momentum but far from robust. This is particularly important, because in non-CF populations, energy dense, nutrient poor diets can lead to chronic diseases, like diabetes, which place a significant health burden on the population (7). There is an increasing prevalence of overweight and obesity among individuals with CF, even among individuals with CF who are pancreatic insufficient and who have severe CFTR mutations (2). The shift toward over-nutrition in CF is likely not only rooted in improved medical therapies, but also high-calorie, nutrient poor dietary intake. Thus, the need for changes in diet quality and recommendations that decrease the risk of chronic disease in the aging CF population are becoming a more urgent need.

This study adds to the limited body of literature that highlights diet quality and the role sources of dietary carbohydrates, fats, and proteins may have on improved glucose homeostasis in adults with CF. Limitations of the study include the small, single center, cross-sectional study design, limiting our ability to infer causality in our findings. Reverse causality is a possibility, where a diagnosis of diabetes or glucose intolerance leads to changes in dietary intake. As an exploratory study, correction for multiple correlations was not performed, thus spurious significant relationships may also have arisen. Planning data was not available to address sample size and power considerations; however, the reported data will inform future prospective studies. It is possible that outcomes were influenced by basal insulin regimens in three participants; however, results involving insulin resistance were similar if these participants were excluded. Further, the current study primarily focused on fasted measures of glucose homeostasis, which may be more reflective of hepatic glucose metabolism and do not represent dynamic changes in glucose tolerance (42). Larger, prospective studies are needed to determine the impact of dietary fats and other macronutrients on glucose tolerance and risk for CFRD using robust, dynamic measures of insulin secretion and sensitivity. If proven effective in the CF population, interventions for diet modification represent a non-invasive and inexpensive measure that could prevent or delay metabolic abnormalities for individuals with CF.

## Conclusion

In conclusion, we showed novel relationships linking the quality of dietary fat, carbohydrates, and protein to glucose homeostasis in adults with CF across the glucose spectrum. While added sugars and dietary glycemic load were associated with metabolic impairment, unsaturated fatty acids and plant proteins were associated with better markers of glucose metabolism. Rigorous clinical trials are warranted to determine if modifying the macronutrient quality of the CF diet will influence glycemic outcomes, such as insulin secretion or insulin sensitivity, and ultimately mitigate decline in glucose tolerance among individuals with CF.

## Data availability statement

The original contributions presented in the study are included in the article/Supplementary material, further inquiries can be directed to the corresponding author.

## Ethics statement

The studies involving human participants were reviewed and approved by Emory University Institutional Review Board. The patients/participants provided their written informed consent to participate in this study.

## Author contributions

TD and JA: initial drafting and finalization of manuscript. BC, P-DN, EI, MB, WH, TZ, and JA: data collection. KE and JA: data analysis. TD, BC, P-DN, PV, MV, AS, TZ, and JA: interpretation of results. All authors contributed to this work, including study conception and design, and reviewed and approved the final manuscript.
