# Supplementary material for: Quality of dietary macronutrients is associated with glycemic outcomes in adults with cystic fibrosis
Source: Front Nutr. 2023 Sep 20;10:1158452. doi: 10.3389/fnut.2023.1158452 (PMC10548231; doi:10.3389/fnut.2023.1158452)
Supplement: Supplementary file 1 [file Table_1.DOCX]

**Supplemental Table 1. Relationships between dietary variables and glucose tolerance outcomes**^1^

| **Dietary Variable^2^** | **Fasting Glucose (mg/dL)** | **Fasting Insulin^3^**  **(uIU/mL)** | **Fasting C-peptide (ng/mL)** | **2-hr glucose^4^** | **HOMA2-IR^3^** |
| --- | --- | --- | --- | --- | --- |
| Total carbohydrates (g) | 0.31 ± 0.22  (0.18) | 0.001 ± 0.001 (0.34) | **–0.01 ± 0.005 (0.01)** | 0.59 ± 0.85 (0.50) | 0.003 ± 0.003 (0.34) |
| Added sugars (g) | 0.29 ± 0.25  (0.25) | 0.002 ± 0.001 (0.21) | **–**0.008 ± 0.006 (0.17) | 1.11 ± 0.84 (0.21) | **0.008 ± 0.003 (0.03)** |
| Glycemic index | **–**1.79 ± 0.96 (0.08) | **–**0.007 ± 0.005 (0.20) | 0.008 ± 0.005 (0.15) | 0.02 ± 3.43 (0.99) | 0.009 ± 0.01 (0.55) |
| Glycemic load | 0.18 ± 0.35  (0.61) | 0.0007 ± 0.002 (0.70) | **–0.02 ± 0.007 (0.04)** | 0.92 ± 1.14 (0.44) | 0.006 ± 0.005 (0.25) |
| Total fat (g) | **–**0.54 ± 0.66 (0.41) | **–**0.002 ± 0.004 (0.59) | **0.04 ± 0.01 (0.002)** | **–5.60 ± 1.86 (0.01)** | **–**0.01 ± 0.009 (0.30) |
| Saturated fat (g) | **–**0.72 ± 1.32 (0.59) | **–**0.001 ± 0.007 (0.86) | 0.04 ± 0.03  (0.18) | –7.06 ± 4.35 (0.13) | **–**0.02 ± 0.02 (0.31) |
| Trans-fat (g) | 2.01 ± 7.15  (0.78) | 0.006 ± 0.04 (0.88) | 0.22 ± 0.16  (0.18) | –13.5 ± 26.7 (0.62) | –0.14 ± 0.10 (0.18) |
| MUFA (g) | **–**1.08 ± 1.60 (0.51) | **–**0.004 ± 0.009 (0.66) | **0.10 ± 0.03 (0.005)** | **–9.51 ± 4.32 (0.046)** | **–**0.02 ± 0.02 (0.37) |
| PUFA (g) | **–**0.88 ± 2.15 (0.69) | **–**0.004 ± 0.01 (0.70) | **0.16 ± 0.04 (0.005)** | –9.3 ± 6.5 (0.18) | **–**0.001 ± 0.03 (0.97) |
| Total protein (g) | **–**0.82 ± 0.68 (0.23) | **–**0.004 ± 0.004 (0.23) | 0.01 ± 0.02  (0.44) | **–**0.96 ± 2.00 (0.64) | 0.009 ± 0.01 (0.36) |
| Animal protein (g) | **–**0.28 ± 0.51 (0.59) | **–**0.001 ± 0.003 (0.64) | 0.009 ± 0.01 (0.46) | 0.60 ± 1.48 (0.69) | 0.01 ± 0.007 (0.08) |
| Plant protein (g) | **–**0.62 ± 0.96 (0.52) | **–**0.004 ± 0.005 (0.44) | **–**0.007 ± 0.02 (0.75) | –3.85 ± 2.54 (0.15) | **–0.03 ± 0.01 (0.04)** |

^1^ Data are adjusted for age and sex and reported as beta estimate ± SE (p-value). Bold values indicate statistical significance (p<0.05). N=26 as dietary data was missing for one subject.

^2^Dietary variables are adjusted per 1000 kcal.

^3^Variable was log10-transformed for analyses.

^4^N=17
